# The Comparative Estimation of Primary Students’ Programming Outcomes Based on Traditional and Distance Out-of-School Extracurricular Informatics Education in Electronics Courses during the Challenging COVID-19 Period

**DOI:** 10.3390/s21227511

**Published:** 2021-11-12

**Authors:** Taras Panskyi, Sebastian Biedroń, Krzysztof Grudzień, Ewa Korzeniewska

**Affiliations:** 1 Institute of Applied Computer Science, Lodz University of Technology, 90-537 Lodz, Poland; sbiedron@iis.p.lodz.pl (S.B.); kgrudzi@iis.p.lodz.pl (K.G.); 2 Institute of Electrical Engineering Systems, Lodz University of Technology, 90-924 Lodz, Poland; ewa.korzeniewska@p.lodz.pl

**Keywords:** primary school informatics, programming outcomes, out-of-school electronics courses, pandemic, crisis-prompted education

## Abstract

The authors decided to investigate the impact of the lockdown period and the resulting limitations in informatics education, especially programming, in out-of-school electronics courses using traditional and distance learning modes in primary school COVID-19 pandemic settings. Two extracurricular courses were held successively; the first electronics course was performed in a traditional out-of-school learning mode using Arduino kits, while the other was held using the TinkerCad circuits virtual environment in distance learning mode. A structured questionnaire was administered to students to map their knowledge of programming. The questionnaire consists of three emotional dimensions: enjoyment, satisfaction and motivation. The fourth dimension was dedicated to the students’ programming outcomes. Three emotional dimensions were addressed to primary school students, while the fourth dimension was addressed to the tutors’ observations toward the students’ programming outcomes. The obtained results revealed that learning modes have no significant impact on students perceiving the programming issues. However, three emotional dimensions revealed a significant difference in the students’ enjoyment, satisfaction and motivation in favor of the traditional learning mode. Our findings are of particular interest in light of possible crisis-prompted distance education in the future but can also serve to inform government institutions and policymakers seeking to develop effective concepts for successful distance learning.

## 1. Introduction

Learning programming is important and essential from an early age. It can help with the development of skills, such as creativity, innovation, problem-solving, logic, algorithmic and computational thinking, which constitute the 21st-century skill-set [1]. Learning informatics at primary school relies heavily on programming. Along with plugged programming lessons, the students’ activities are related to making, tinkering and playing using different educational methods and pedagogical strategies [2]. Besides the schools, talent development is also organized alongside the Polish school educational system. A large number of informatics competitions, contests, summer schools, Olympiads, and extracurricular talent development programs for highly motivated students are available. A large number of traditional competitions, including national Olympiads or competitions for young researchers and engineers, which are mainly supported by the Ministry of Education could boost and accelerate the informatics skills of talented students [3]. However, due to the COVID-19 pandemic, all primary school students are forced to stay at home, and teachers, headmasters and organizers of out-of-school activities changed the formula of the previously traditional informatics education to the version with the use of the Internet. The closure of schools as a result of COVID-19 has been a critical global incident from which to rethink how nowadays distance informatics education works in Poland.

Our study concerns the primary Polish informatics education in the times of the COVID-19 virus pandemic, which forced everyone to operate in a different model, mainly due to the change in the way most educational institutions functioned: from traditional to distance. Distance education (and even wider use of information and communication technologies), which was only an option a few months ago, became a necessity. Currently, it is the only way of conducting programming educational activities available to primary school students. It is worth noting that distance education before the pandemic can only partially help us act meaningfully today. First, because earlier such distance education was most often the choice of the school audience. Those who have benefited from its were mainly primary school students broadening their qualifications via out-of-school and/or extra-curriculum courses or workshops. The target audience also included students living in remote locations who were unable to participate in traditional education. Finally, distance education has been used at home and as a complement to traditional school programming lessons. In all these cases, we deal with a situation where the student or his parents have decided to use distance education benefits. In the new conditions, we have a compulsion, which means that regardless of the possibilities, competencies and willingness, such solutions must be used by everyone, not only those who want and can. Moreover, the crisis-prompted primary school informatics education reflects the shift from existing formal education, both traditional and out-of-school, to distance. This means that all forms of traditional education nowadays (during a pandemic) becomes distance only.

In this article authors analyzed the selected available research studies dedicated to crisis-prompted distance informatics education. Next, the authors included first experiences of distance education activities in the new pandemic situation and attempted to compare the students’ programming learning outcomes achieved via the traditional and distance out-of-school electronics courses. Thereafter, a range of issues that should be addressed was outlined and structured. Finally, the authors made a set of recommendations for further research in particular and suggestions for further development of Poland’s primary out-of-school distance informatics education in general.

The authors decided to investigate the impact of the pandemic period and the resulting limitations in out-of-school distance informatics education of primary school students. In this aspect, it became purposeful to pose crucial research questions:

1. Does the pandemic influence the primary students’ programming learning outcomes in out-of-school extra-curriculum settings?
2. Do the basic emotions (satisfaction, enjoyment and motivation) have a crucial impact on the primary students’ programming learning outcomes during the COVID-19 distance education?

## 2. Distance-Blended-Traditional School Education

Many studies have been conducted to evaluate distance, blended and traditional learning student outcomes to convince critics of the appropriateness and effectiveness of choosing a particular form of education. Some authors confidently claimed that “nothing can replace the face-to-face interaction between teacher and students” [4] and favor traditional classroom instruction, stating “online learners will quit more easily” and “online learning can lack feedback for both students and instructors” [5]. Other researchers who aver distance education produces students who perform better than their traditional classroom counterparts [6,7], while the rest select the status quo, arguing that there is no significant difference in test scores, assignments, participation grades and performance between distance and traditional classroom students [8,9,10,11].

Among two distance and traditional educational “milestones”, some researchers and educators try to find a grain of truth in other alternative forms of teaching/learning. Thus, taking the best from distance education and its concepts [12] (e.g., online learning, virtual learning, distance education, m-learning, massive open online courses (MOOC), learning management systems) and from traditional “old fashioned” (e.g., direct instructions and lectures, seatwork, group activities, “chalk and talk”) coated by “modern” (e.g., inquiry-based learning, problem-based learning, game-based learning, collaborative learning, activity-based learning) teaching methods and approaches, in the current digital era, the blended learning has been actively promoted by researchers and profiled by educators. Obviously, blended education has its supporters, admirers and followers simultaneously with its opponents and critics among school staff, educators and researchers. Several earlier studies confirmed that blended learning, compared to traditional, increased positive effect on the students’ achievements [13,14,15], while others claimed that blended learning had no significant effect [16,17,18]. Some authors showed a significant improvement in the students’ learning outcomes after the application of the blended learning approach [19,20,21], while others had different opinions supported with their findings [22,23].

Recent research [24] has shown that distance students have worse outcomes than full-time students in informatics assignments. However, other authors [25] noticed a great perception of students during the programming course while peer-reviewing their codes in an online environment. Moreover, the authors [26] found that students participating in online peer assessments performed better in terms of their programming skills as compared to those students who were only exposed to traditional teaching and learning approaches. The researchers [27] emphasized that most of the games cover basic programming concepts, such as sequence commands, simple and nested if statements, loops or functions, and could be integrated into distance informatics education. The author [28] made the comparison of academic performance of students in an online vs. traditional engineering course, where they noticed that essential continuous efforts should be made to improve online pedagogy.

The advantages and disadvantages of distance–blended–traditional instructional modalities need to be fully fleshed out and examined to truly determine which medium generates better student performance. These educational methods have been proven to be relatively effective in the outlined conditions at the corresponding external and internal factors, but the question to be asked is if one is truly better than others in all educational dimensions and learning aspects at primary school settings. Moreover, new information and communication technologies (ICTs) present important opportunities for augmenting the benefits in the context of each chosen form of education as they can also be used to incrementally build educational resources that can be revisited by teachers and students throughout their studies. Despite previous reports on the comparison of traditional and distance learning, the evaluation of the students’ programming outcomes that are delivered through traditional learning methods compared to crisis-prompted online learning has not been widely available. The majority of studies on distance learning methods reported student perceptions of programming made before the pandemic came. Student feedback could provide important information for the evaluation of distance learning so as to improve future learning strategies. Therefore, the study aimed to analyze primary school students’ perception of programming in traditional and distance out-of-school learning modes and the impact of emotions on educational outcomes.

## 3. Informatics Lessons during the Pandemic

The COVID-19 pandemic contributed to a rapid change in the perception of school education and the role of distance learning in the teaching process. Polish schools had to face many problems, doubts and issues that arose not only when conducting distance lessons but also during the development and implementation of distance learning methods by schools. When conducting remote classes in informatics, one of the main problems was the selection of appropriate tools enabling the effective implementation of the lessons. Due to the lack of clear guidelines regarding the choice of software and the method of conducting lessons, teachers used Google Classroom, Webex, or Moodle, using this tool to implement remote classes in an asynchronous manner or Zoom, MS Teams in synchronous teaching. As part of the informatics classes, teachers could record and publish short guide films on the use of programs, implementation of tasks, or explaining problematic issues related to the topic of the lesson. Moreover, because students are often accustomed to informal interactions during lessons, running informatics virtually has meant accepting how students prefer to communicate: chat, emojis, memes, and slang. Some teachers also tried to adapt remote lessons, both for students using desktops and laptops and for students who only had the opportunity to participate via mobile devices, such as smartphones or tablets [29]. However, access to ICT devices was only one of the factors affecting the ability to establish remote communication between the teacher and the student [30]. The teachers “thrown into the deep” felt lonely and deprived of help. Overnight, they had to switch to new, often unknown methods of work. A huge problem was the time-consuming preparation of distance informatics teaching [31]. The most common technique, used especially at the beginning of the pandemic, was emailing tasks to be done, chapters to read in textbooks or instructions to read from the Internet. Even these simple messages initially caused a lot of difficulty for students due to the chaos in using different platforms and messaging by teachers and the huge number of notifications they sent about activities and tasks to be performed. The students had trouble finding and executing them according to the given deadline [32]. Both groups—teachers and students—reminded each other of their low digital competencies. Teachers claimed that even in the older grades of primary school, there were children who were unable to pick up email or access e-learning platforms. On the other hand, students claimed that some teachers lacked the basic knowledge to work at the computer [33]. One of the reasons why teachers flooded their students with numerous tasks, used frequent tests or quizzes was the need to complete the Core Curriculum and give grades. Both overloaded programs and teaching methods weaken the motivation to learn informatics [34]. Distance informatics education has not achieved high marks in any group of people involved—teachers, students or parents [35]. The vast majority want to return to school. The reasons are difficulties with the ICT equipment, time consumption, lack of direct contact and low effectiveness of distance informatics learning.

The current school in Poland, despite continuous reforms, does not keep up with the broadly understood didactic and educational goals. Its didactic and educational activity to date, conducted mainly during school hours, using traditional methods, with a small number of extracurricular activities, cannot provide students with comprehensive development of their personality. Moreover, during a pandemic, frequently changing regulations and the dynamic epidemiological situation generates many unknowns. Extracurricular out-of-school activities are an extension of the didactic and educational school process, giving students the opportunity to satisfy, develop and deepen their interest and creative work in informatics-related fields. As a result, extracurricular activities that properly fulfill their intended functions may have a greater impact on students than crisis-prompted distance school informatics lessons. The extra-curriculum out-of-school activities are relatively easy with informatics, in particular, programming, which has been implementing distance educational resources and learning management systems for years, such as virtual classrooms, PowerPoint presentations, online compilers and whiteboards, etc. However, the extracurricular lessons of electronics, mechatronics and robotics have always depended more on the face-to-face teaching dynamics, as they require a more hands-on approach, specialized equipment and installations, materials for manual work, and, ideally, direct interactions. Due to the described situation, the authors focused on students’ programming outcomes in extracurricular electronics out-of-school courses.

## 4. Extracurricular Courses

Two extracurricular courses were held successively. The first was dedicated to informatics, in particular electronics, performed in traditional/stationary out-of-school education using Arduino kits. The other was held using TinkerCad circuits of the Arduino virtual environment in distance teaching mode.

The traditional Arduino course was held at the Institute of Applied Computer Science at the Lodz University of Technology, using the Arduino open-source platform as the main hardware. Arduino is essentially a computer built into a single chip, and its brain is a microcontroller. There is a variety of Arduino boards available with different shapes, sizes and capabilities. The traditional electronics course was taught using the Arduino Uno board in conjunction with the mBlock, an open-source visual-based programming environment. Students were able to write programs by dragging and dropping building blocks in their already usual and habitual way. Furthermore, the mBlock interface allows one to control a variety of Arduino-based programmable electronic projects. The main advantages of Arduino over its competitors are the expendability, price and sizes [36]. Apart from the price, what is crucial is the sensing capabilities of the Arduino platform. Thus, the advantage of Arduino lies in its flexibility and endless possibilities of its usage, enabling students to program it according to their needs. Last but not least, the students could switch from a block-based mBlock visual drag-and-drop environment to a more sophisticated level of programming using the Arduino IDE text-based environment in their future personal development in the programming domain. Dickes and Farris [37] emphasized that the reproduction of visual blocks in a text form leaves out an important element of the experience of programming, such as “seeing the same code from different perspectives”.

The distance TinkerCad circuits course was organized by the Institute of Applied Computer Science at the Lodz University of Technology on the online MS Teams communication and collaboration platform. TinkerCad is a free software application or simulator that can be used without having to first download and install it on a computer or smartphone. Developed by AutoDesk, it is a cloud-based software that is a virtual representation of a real-world Arduino circuit. TinkerCad allows students to design circuits, program micro-controllers and incorporate the virtual electronics directly into their projects. TinkerCads’ circuit simulator allows students to arrange their Arduino circuits in an easy “drag and drop” Scratch-based environment and test them virtually without having to make them. Moreover, the editor designed by the TinkerCad allows students to view blocks and text code in C/C++ side by side to make it easier to transition from visual to text-based programming.

In both traditional and distance extracurricular courses, students were able to design and program Arduino-compatible devices with multiple sensors, such as temperature and humidity, light, movement, etc., to control LEDs and LED bars using dimmer switches, using a push button to switch LEDs, RGB LEDs, to output text information on an LCD display, to program a LED matrix, to create the air pollution monitoring system, or to design and program a radar using a servo motor and an ultrasonic sensor [38]. Moreover, both extracurricular courses aimed at teaching programming principles, such as loops, synchronization, variables, conditionals, operators, broadcasts, functions and more [39], through the use of graphical blocks which overlapped by the “drag and drop” technique.

## 5. Course Organization and Participants

Both extracurricular courses consisted of 10 sessions presented in Table 1, each lesson taking 2 lesson hours (1.5 clock hours). The organizer did not establish a baseline, and no pre-tests were performed in any of the groups; that is, participants could be both novice and programmers with some background experience in visual block-based environments. However, no participant declared prior programming knowledge in Arduino or TinkerCad environment.

**Figure 1:**
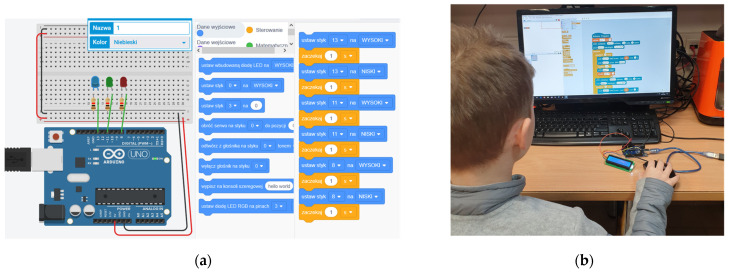
The illustration of students performing programming tasks in electronics courses in different learning modes (**a**) An example of the task from the second session using the distance form of extracurricular primary school education; (**b**) An example of the task from the eights session using the traditional form of extracurricular primary school education.

The traditional Arduino course sessions were held only on weekends, that is, every Saturday and Sunday. Parents were obliged to collect their children after course sessions. Each participant in a group was working on their personal desktop computer place (see Figure 1b). Before the start of a new course edition, the enrolled participants could choose, for convenience, to work on a computer or a tablet. Each computer place for a particular participant remained the same until the end of the traditional Arduino course. The Arduino course duration was three months (from December 2019 until March 2020), and the session was provided each week. The group was formed by 21 students aged 12 to 13 years.

The distance TinkerCad course sessions were held on weekends but also on workdays, at the MS Teams online platform. Participants, who had logins and passwords, could log in to TinkerCad classes on their own. The key requirement of the TinkerCad course was to work on the appropriate equipment, i.e., each of the participants had to have prepared either a computer or a laptop. Mobile phones and tablets were excluded as ICT equipment as not appropriate for an online electronics course. The TinkerCad course duration was three months (from March 2020 until June 2020), and the session was provided each week. The group was formed by 23 students aged 12 to 13 years. The interested students used an online form for registration purposes for both traditional and distance extracurricular electronics courses. The registration relied on submitting basic information, which included the students’ first and last name, age, and email.

Four tutors were always engaged in teaching one session and directing the whole group of participants regardless of the course. Tutors provided strategies to assist participants in remembering, understanding and organizing the presented information better. Each session started with the previously presented material and offered an opportunity for reinforcement. All tutors had sufficient pedagogical competencies in using different educational approaches in programming and STEM courses. Tutors were usually Ph.Ds. in computer science or engineering with a minimum of 7 years of experience in teaching classes in visual programming. They had competencies related to planning meaningful session topics and programming activities within the particular course curriculum. Tutoring competencies also refer to guiding participants in the learning process during the course sessions, including applying motivation techniques, personalizing activities, and regulating the degree of participant commitment with flexibility and efficiency.

This section may be divided into subheadings. It should provide a concise and precise description of the experimental results, their interpretation, as well as the experimental conclusions that can be drawn.

## 6. Data Collection

A structured questionnaire designed by tutors was administered to students to map their knowledge, use and attitudes toward the programming. The questionnaire consisted of three crucial emotional dimensions: enjoyment, satisfaction and motivation. The fourth dimension was dedicated to the programming outcomes displayed using a set of validation criteria. The research was transversal, i.e., it covered a sample (44 students) at a particular moment.

According to [40], developing programming skills simultaneously develops logical and algorithmic thinking, creativity, computational thinking and a systematic approach to problem-solving. Nevertheless, the authors focused only on programming learning outcomes emphasized by the conceptual and cognitive difficulties in dealing with the process of constructing block-based programs and also challenges posed by specific programming constructs, such as variables, various types of looping structures, logical flow using conditionals and Boolean logic.

There are numerous articles that describe the role of enjoyment, satisfaction and motivation in traditional, blended, and distance education [41,42,43,44,45,46,47,48,49,50]. Nevertheless, little work has examined how the students’ learning outcomes toward programming in distance/traditional educational teaching modes would change in pandemic settings. Only now, amid COVID-19, the authors were able to explore enjoyment, satisfaction and motivation on students’ programming outcomes in a distance crisis-prompted extracurricular electronics course and compare it to the traditional one. Moreover, the authors did not explore the students’ intrinsic perceptions and attitudes as the determinants of their behavioral intention but only the significance of relationships between the students’ programming outcomes and perceived enjoyment, satisfaction and motivation in different educational learning modes.

In order to collect data on the students’ programming experiences, a questionnaire strategy was deemed most appropriate. Data were collected by means of ‘asking questions’ in an online-based self-completion questionnaire Q1–Q15 containing closed questions (see Table 2). The questionnaire was distributed at the end of the distance/traditional electronics course. All 44 students voluntarily completed the questionnaire.

The questions addressing the participants’ distance/traditional learning attitudes were adopted from the scales developed by authors of [51,52,53,54,55,56]. These instruments measured the students’ satisfaction, enjoyment and motivation. The applied questions were modified in accordance with the educational teaching mode and extracurricular learning settings. The questions were divided into three main dimensions: the Q1–Q5 are dedicated to the students’ satisfaction, the Q6–Q10—the students’ enjoyment, and Q11–Q15—the students’ motivation.

The fourth dimension was addressed to the tutors’ observations toward the students’ programming outcomes. Every tutor evaluated the students’ programming outcomes with corresponding validation criteria PO1–PO5 in different educational teaching modes. These criteria were adopted from the Polish National informatics curriculum applicable in the didactic process in the primary school.

For each question (Q1–Q15), the students responded to a self-referring statement on a five-point Likert scale ranging from “1 = definitely disagree” to “5 = definitely agree”. Each criteria PO1–PO5 was also marked by the tutors based on the same five-point Likert scale.

## 7. Result and Discussion

### 7.1. Descriptive Statistics

The descriptive statistics presented in Table 3 include the short names for each dimension, the mean, standard deviation, skewness and kurtosis of the students’ answers and the tutors’ criteria toward the traditional and distance educational teaching/learning mode. According to Bai and Ng [57], data are normally distributed when skewness and kurtosis are respectively within the range of ± 1 and ± 3. Table 3 shows that the distribution of the data of all dimensions was close to a normal distribution.

The analysis of the overall questionnaire in all three dimensions Q1–Q15, revealed the highest mean score was in questions Q11—4.52 within the traditional mode and Q8—4.24 within the distance learning mode. The lowest mean score within the particular dimension was shown in question Q4—3.57 in the traditional mode and question Q10—3.33 in the distance mode, respectively. The pairwise analysis of the mean scores of presented learning modes conducted for the questions Q1–Q15 discovered the superiority of traditional over distance mode in all questions except questions Q8 and Q14. Moreover, question Q2 revealed the biggest difference—0.95 in favor of the traditional mode, and question Q14—the smallest difference—0.02 in favor of the distance learning mode. However, to gain crucial insights into the differences in educational learning modes, each particular dimension should be discussed in greater depths.

The highest mean score in the satisfaction dimension toward the educational learning mode was shown in question Q2—4.43 in traditional and in question Q5—4.05 in distance mode. The lowest mean score for the traditional learning mode was reported in question Q4—3.57 and in the distance learning mode in question Q2—3.48. The pairwise analysis of the mean scores of traditional and distance educational learning modes in the satisfaction dimension revealed the biggest difference in question Q2—0.95. This question is related to the possibility of the choice of Arduino or TinkerCad programming tool in the future. The smallest difference was reported in question Q4—0.05. The Q4 question is related to a positive feeling about the students’ self-fulfillment and happiness in the electronics course.

In the enjoyment dimension, within the learning mode, the highest mean score was obtained in question Q9—4.22 in traditional and in question Q8—4.24 in distance learning mode. The lowest mean score was dedicated to question Q10 for both traditional—3.86 and distance—3.33 learning modes. Moreover, questions Q10 and Q9 showed the biggest differences between the pairwise comparisons of the traditional and distance learning modes in the enjoyment dimension—0.53 and 0.60, respectively. The smallest difference was shown in question Q8 in favor of distance learning mode—0.07.

In the third dimension—motivation, the highest mean score was obtained in question Q11—4.52 within the traditional and in question Q14—3.76 within the distance learning mode. This question is crucial as high motivation is expressed through enjoyment, interest, self-efficacy that becomes an important determinant of a student’s learning intention and, as a result, could significantly influence their future success [44]. Simultaneously, the smallest difference between the mean scores among all Q1–Q15 questions was shown in question Q14—0.02 of the motivation dimension. The pairwise comparison of learning modes’ mean scores showed the above-mentioned Q14—0.02—the smallest difference in favor of the distance learning mode in the motivation dimension in particular and the overall questionnaire in general. The biggest difference was reported in question Q11—0.81 in favor of the traditional learning mode, which was the second biggest in the Q1–Q15 questionnaire.

The last dimension, dedicated to the tutors’ observations, noted the lowest mean score within the traditional learning mode among all Q1–Q15 questions and PO1–PO5 criteria obtained in criterion PO3—3.43. The pairwise analysis of educational learning modes showed the difference between the mean scores fell within the range between 0.02 and 0.22 in the “programming outcomes” dimension. Moreover, despite the learning mode, the analysis revealed almost identical mean scores in criteria CR2–CR5. The difference was uniform throughout the criteria PO1–PO5, with a slight downward toward the distance learning mode.

The overall results showed that all students had a significantly high mean score when answering all questions (see Table 3). Moreover, the descriptive statistics also revealed the high level of programming outcomes regardless of the teaching mode. Generally, the majority of the mean scores of each particular student answer and evaluation criterion was higher than 4 in traditional teaching mode, which indicates the Likert mark—“agree”. However, in distance teaching mode, the majority of the mean scores was higher than 3, which indicates the Likert mark—“Undecided/Hard to say”. For a more detailed study of the difference between the teaching modes and their impact on every dimension, the Student’s *t*-test was carefully applied.

### 7.2. Statistical Analysis

Differences between the two teaching/learning modes were analyzed using Student’s *t*-test. All tests were two-tailed. *P* < 0.05 was considered statistically significant. All analyses were conducted using the Statistical Package for the Social Sciences (IBMSPSS, version 21). Analysis of internal consistency showed that the overall Cronbach alpha obtained for the Q1–Q15 questions was 0.79. The values of Cronbach’s’ alpha of the four dimensions ranged from 0.74 (enjoyment) to 0.83 (motivation), indicating good scale reliability.

Table 4 shows the results of the *t*-test conducted for all students’ Q1–Q15 answers and tutors’ PO1–PO5 evaluation criteria. A significant difference was found between students regarding their satisfaction, enjoyment, motivation and programming outcomes toward the learning mode.

Table 5 shows the difference between the tutors’ evaluation criteria PO1–PO5 regarding the students’ programming outcomes toward the learning mode. No significant differences were found between students regarding their programming abilities. The learning modes (distance or traditional) had no significant impact on students perceiving the programming issues and performing the engineering tasks. The received results indicate that the overall significant difference between teaching/learning modes presented in Table 4 did not reflect the students’ significant differences in programming outcomes (Table 5). Moreover, if the students’ programming outcomes perceived in electronics courses in the distance and traditional teaching/learning modes did not differ significantly, the other three dimensions (satisfaction, enjoyment, motivation) should have a great impact on the overall results. It raises the question “what dimension has the biggest impact on students perceiving programming knowledge in different teaching/learning modes?”

Table 6 shows the difference between the students’ answers Q1–Q5 regarding the satisfaction dimension toward the learning mode. No significant differences were found in questions Q1, Q4 and Q5. However, significant differences were revealed in questions Q2—(*p* = 0.000) and Q3—(*p* = 0.012). Question Q2 is related to the statement that “I will continue to choose the (TinkerCad distance/Arduino traditional) as a learning tool in the future”, where students more strongly agreed with this statement in traditional (4.43 ± 0.66) than in distance (3.48 ± 0.75) learning modes. Question Q3 is related to the statement: “I would recommend the (TinkerCad distance/Arduino traditional) to other students as a learning tool”, where students preferred to agree with this statement and recommend the Arduino traditional (4.22 ± 0.60) more often than TinkerCad distance (3.61 ± 0.86) extracurricular courses. In the satisfaction dimension in distance learning mode, students did not want to recommend the TinkerCad tool and to continue programming activities using TinkerCad in the future which radically distinguished their answers from students in the traditional learning mode, which used Arduino equipment for programming purposes. The increased level of interactivity now possible with computer games and with the communication features of the Internet, in conjunction with home-schooling, has heightened both the promise of greatly enriched learning and the concerns related to increased risk of harm [58]. During the COVID-19 pandemic, students were overloaded with various virtual activities that tended to focus on issues of access and the amount of time they were spending with the new educational tool and were juggling an ocean of resources and measures to cope with the new routines. Students in distance learning mode experienced digital fatigue, overwhelmed by media, resources and messages, but also priorities of ‘digital’ pedagogies. Moreover, students felt exhausted, anxious and stressed by using various ICT devices, as a result, they could perceive the technostress [59]. Technostress is associated with psychological and behavioral disorders and impairment of work and life satisfaction, leading to reduced productivity [60]. Therefore, despite the availability and simplicity of the TinkerCad programming tool, students accepted, learned and analyzed it, but some of them would only use it in further informatics education reluctantly.

Table 7 shows the difference between the students’ answers to Q6–Q10 regarding the enjoyment dimension toward the learning mode. No significant differences were found in questions Q6 and Q8. However, significant differences were noted in questions Q7—(*p* = 0.037), Q9—(*p* = 0.030) and Q10—(*p* = 0.039). In the enjoyment dimension, unlike satisfaction, three questions showed significant differences in student responses toward the learning mode. Question Q7 is related to the statement: “The (TinkerCad distance/Arduino traditional) has caused my interest in learning programming”, where students more strongly agreed with this statement in traditional (4.04 ± 0.71) than in distance (3.58 ± 0.75) teaching modes. The trend remained the same for questions Q9 and Q10, where the first is related to the statement: “To see the advance of my project in (TinkerCad distance/Arduino traditional) produced a positive emotion” (4.22 ± 0.67 vs. 3.62 ± 0.97) and the second: “This (TinkerCad distance/Arduino traditional) course had risk-taking elements which I enjoy” (3.86 ± 0.76 vs. 3.33 ± 0.91) in favor of traditional learning mode. Question Q7 in the enjoyment dimension demonstrated that the TinkerCad tool in distance learning mode did not facilitate interest to learn programming. In Question Q9, students indicated a better self-rating and demonstrated individual attitudes toward the appearance of positive emotions in the traditional Arduino course compared to those who reported a poor self-rating of their capacities to experience joy, interest, contentment in the TinkerCad distance extracurricular electronics course. According to [61], “positive emotions transform people for the better, giving them better lives in the future”. Moreover, positive emotions could broaden students’ thought–action repertoires, undo lingering negative emotions, fuel psychological resilience and enhance emotional well-being. Therefore, positive emotions play a crucial role in the educational domain in primary school settings. At the beginning of the distance learning mode, students’ emotions were positive but gradually changed toward struggle with self-efficacy, mental health issues, digital fatigue, cyberbullying, online abuse and numerous other consequences and e-threats of crisis-prompted distance education [62,63,64]. In Question Q10, the students’ self-rating was straightforward: the TinkerCad tool did not possess risk-taking elements in comparison with the Arduino equipment. All TinkerCad components are virtual and absolutely safe to use because it uses no physical, electronic components. However, students at the Arduino traditional course could be involved in undesirable reactions of electronic circuits due to the students’ mistake in building and/or programming: shorting I/O pins to ground, applying overvoltage to I/O pins, burn-out LEDs, breaking the wire, etc. These mistakes could lead to the fault, failure or even destruction of Arduino circuits (burning smell, the scorch mark on a component, or the error message).

With respect to the motivation dimension, no significant differences were found in questions Q12–Q15. However, a significant difference was demonstrated in question Q11—(*p* = 0.003). The motivation dimension revealed the significate difference only in one question. Question Q11 is associated with the statement that “The (TinkerCad distance/Arduino traditional) can arouse my motivation to learn the programming language”, where students in the traditional learning mode more strongly agreed with this statement (4.52 ± 0.59) than in the distance (3.71 ± 0.95) learning mode. The Arduino traditional course motivated students to learn programming definitely more than the TinkerCad virtual tool. Distance learning in comparison to regular face-to-face lessons is characterized by greater flexibility in scheduling, managing time and tasks, the opportunity to individualize and control learning processes, the potential to enhance self-regulated learning programming skills and the easy distribution of information [65]. However, it places high demands on the learners’ ability to regulate their learning and motivation and thus poses an increased risk of digital fatigue and passive procrastination. According to authors [66], motivation remains one of the crucial problems of teaching/learning in crisis-prompted distance education. Martin [67] mentioned motivation as one of five important things every teacher should remember while using distance education tools. Hence, the TinkerCad distance extracurricular course was also shown to correlate with lower student motivation, where the Arduino traditional course plays an important role in the tendency to enhance student motivation in learning programming.

The migration to a new distance learning space has faced several major concerns relating to students’ technological and psychological and emotional factors [68]. With respect to the technology factor, students who already utilized digital learning platforms had fewer barriers to distance education compared with students who did not have ICT devices and the Internet at home [69]. Nevertheless, according to GUS and OECD study [70,71], over 90% of Polish households have an Internet connection at home, which is above the world average. Another GUS report [72] revealed that almost 84% of Polish households had at least one computer. Moreover, the finding described in the Librus study [73] provided during the pandemic in 2020 indicated that almost 92% of students had access to computers and 72% to mobile phones. During the pandemic crisis, the majority of Polish primary school students were fully supplied with ICT devices for distance informatics learning. Nowadays, the new digital generation of students was born into a world of information technology with an Internet connection and hands-on experience [74]. Therefore, the access and use of ICTs have not been the crucial challenges posed by COVID-19 pandemic in Poland’s education system. However, with respect to emotional factors, the obtained results revealed that students self-rating of satisfaction dimension in questions Q2 and Q3 (see Table 6), enjoyment dimension in questions Q7, Q9 and Q10 (see Table 7) and motivation dimension in question Q11 (see Table 8) were significantly associated with their attitudes toward the learning mode. According to authors of [75], after the prolonged crisis-prompted cognitive mobilization and without any substantial cognitive gain, a cognitive exhaustion phase appears that could lead to motivational and emotional dysfunctions in students’ educational development. Therefore, emotional factors play an important role in distance education and could negatively influence students’ programming learning outcomes. On the other hand, tutors’ competencies, programming skills, creativity, flexibility in scheduling and student–tutor interactions were positively interrelated with student programming learning outcomes in the distance and traditional learning modes in extracurricular out-of-school settings (see Table 5).

The presented results are in line with the MEJOREDU report [76] provided in a Mexican setting, where students reported difficulty, stress and frustration followed by lack of motivation expressed as laziness, tiredness and boredom during the pro-longed distance crisis-prompted education. Moreover, the study conducted in Belgium [77] and the US [78] indicated a negative impact of distance learning on children’s self-motivation and satisfaction. However, concerning the last dimension, the presented results contradict previous studies performed in the Netherlands, United Kingdom, and Switzerland [79,80,81], where researchers found a significant decrease in the students’ performance in the distance learning mode.

## 8. Conclusions and Future Work

The study presented the differences in crisis-prompted and traditional learning modes in Polish primary school students in extracurricular electronics courses. In both courses, the authors intentionally used programming that required the students to design electronic circuits with multiple sensors in a visual block-based environment without handling the complexity of the text-based programming syntax. Thereby, students were enabled to conduct data analyses using sensor data. The advantage of using such data in informatics education is the shift of focus from just processing and analyzing data to the whole data life cycle from their acquisition, modeling, programming through processing and analysis to visualization. Moreover, processing sensor data and events in both electronics courses were central from a technical perspective but have not been discussed enough in the Polish informatics Core Curriculum in primary school.

Students’ attitudes to particular learning modes were grouped into four basic dimensions: enjoyment, satisfaction, motivation and programming outcomes. The questions from the first three dimensions were addressed to the students’ learning attitudes, while the last dimension with included evaluation criteria was addressed to the tutors. Analysis of each dimension separately presented no significant differences in the students’ programming outcomes. The learning modes (distance or traditional) had no significate impact on students perceiving the programming issues and performing the engineering tasks. Howsoever, our results also indicated the significant difference in questions concerning enjoyment, satisfaction, motivation dimensions that comprised a significant contribution to the overall results. Our findings are of particular interest in light of possible crisis-prompted distance education in the future but can also serve to inform government institutions and policymakers seeking to develop effective concepts for successful distance learning.

Several areas of interest could be addressed in future research. Other important dimensions (e.g., self-efficacy, creativity, intention, self-awareness) should be approached to gain a clearer understanding of crisis-prompted distance informatics education from different perspectives. A longitudinal design would allow for insights into changes in students’ perceived programming outcomes by means of self-regulated learning, providing further information about the underlying mechanisms that influence distance learning success in primary school settings. Our study focused on students’ subjectively perceived enjoyment, satisfaction and motivation with objectively perceived tutors’ observations toward the students’ programming outcomes. Future research could aim to incorporate other measures of students’ learning success (e.g., grades, time per task, code transparency and readability). Our study focused on two groups of students, namely one with a traditional learning mode and the other with a crisis-prompted distance learning mode. Future research could be addressed to the third group of students with a blended/hybrid learning mode. Finally, the impact of other factors, such as parents’ support, well-being, integration and other economic and social factors, should be investigated in future studies on primary Polish informatics education.

## Figures and Tables

**Table 1:** Course organization divided into sessions.

Sessions	Electronic Equipment	Exemplary Tasks
1	Participants are introduced to the Arduino Mblock/TinkerCad environments.	Each student should have a successfully running LED blink circuit made from scratch.
2	Arduino Uno board, breadboard, wires, LEDs.	Each student should create a program using multiple LEDs blink circuits (red, green, yellow) for the correct traffic lights’ simulation with and without using variables (see Figure 1a).
3	LEDs and push buttons.	Each student should create a program to toggle the LEDs using the push button (pressed/unpressed).
4	LEDs, RGB LEDs and potentiometer.	Each student should create a program to control the LEDs using the potentiometer and resistor.
5	LEDs, PIR motion and ultrasonic distance sensors.	Each student should create and program a distance controlling system. The LEDs should switch on gradually in accordance with the approach of the object to the sensor.
6	LEDs, temperature sensor and photoresistor.	Each student should create and program a temperature controlling system. The LEDs should switch on gradually in accordance with temperature.
7	7-segment display.	Each student should create a program that will display in the decimal system, every second, the time that has elapsed since the program was started on the 7-segment display.
8	LCD display and temperature and distance sensors.	Each student should create a program that will display the temperature and humidity on the LCD display (see Figure 1b).
9	8 × 8 LED matrix.	Each student should create a program that will light up the LEDs on the LED matrix in the checkerboard pattern.
10	8 × 8 LED matrix and joystick	Each student should create a classic snake game using a LED matrix and a joystick.

**Table 2:** The anonymous questionnaire directed to the students to receive feedback on satisfaction, enjoyment and motivation dimensions and to tutors to receive feedback on the students’ programming outcomes.

Satisfaction (Directed to Students)	Short Name
I really like to learn through (TinkerCad distance/Arduino traditional), and I hope I have the opportunity to practice more.	Q1
I will continue to choose the (TinkerCad distance/Arduino traditional) as a learning tool in the future.	Q2
I would recommend the (TinkerCad distance/Arduino traditional) to other students as a learning tool.	Q3
The (TinkerCad distance/Arduino traditional) project’s success makes me feel a sense of accomplishment.	Q4
I liked that there was a (TinkerCad distance/Arduino traditional) activity in each period of the course.	Q5
**Enjoyment (Directed to Students)**	
Learning with the (TinkerCad distance/Arduino traditional) makes me feel happy.	Q6
The (TinkerCad distance/Arduino traditional) has caused my interest in learning programming.	Q7
It was a pleasure to learn programming in (TinkerCad distance/Arduino traditional).	Q8
To see the advance of my project in (TinkerCad distance/Arduino traditional) produced a positive emotion.	Q9
This (TinkerCad distance/Arduino traditional) course had risk-taking elements, which I enjoy.	Q10
**Motivation (Directed to Students)**	
The (TinkerCad distance/Arduino traditional) can arouse my motivation to learn the programming language.	Q11
The design of the (TinkerCad distance/Arduino traditional) projects enhances its attractiveness to learn programming.	Q12
I am confident I will do well in programming in the future.	Q13
I will put enough effort into learning programming.	Q14
Knowing programming will give me a career advantage.	Q15
**Students’ Programming Outcomes (Directed to Tutors)**	
The student has the ability to determine what variables are required in a program to achieve the goals of the task.	PO1
The student describes how a variable changes values in a loop using repeat until, repeat and forever blocks.	PO2
The student understands what a nested loop is and is able to develop programs that use nested loops.	PO3
The student knows how to use logic operators, including Boolean expressions, in a programming context.	PO4
The student knows how to use if-then-else conditional statements in control blocks to achieve the goals of the task.	PO5

**Table 3:** Descriptive statistics of the analyzed questions.

Short Name	TinkerCad Distance				Arduino Traditional			
	Mean	Std. Dev	Skewness	Kurtosis	Mean	Std. Dev	Skewness	Kurtosis
Q1	3.71	0.78	−0.11	−0.16	4.17	0.78	−0.32	−1.22
Q2	3.48	0.75	−0.30	−0.07	4.43	0.66	−0.77	−0.35
Q3	3.61	0.86	−0.15	−0.38	4.22	0.60	−0.09	−0.20
Q4	3.52	0.87	0.17	−0.48	3.57	0.66	0.77	−0.35
Q5	4.05	0.74	−0.07	−1.04	4.26	0.69	−0.39	−0.72
Q6	3.43	0.87	−0.01	−0.47	3.91	0.73	0.14	−1.01
Q7	3.58	0.75	0.13	−0.09	4.04	0.71	−0.06	−0.82
Q8	4.24	0.62	−0.19	−0.36	4.17	0.65	−0.18	−0.46
Q9	3.62	0.97	−0.19	−0.79	4.22	0.67	−0.28	−0.63
Q10	3.33	0.91	0.55	−0.25	3.86	0.76	0.23	−1.14
Q11	3.71	0.95	−0.12	−0.86	4.52	0.59	−0.81	−0.22
Q12	3.62	0.67	0.63	−0.50	3.82	0.65	0.18	−0.46
Q13	3.43	0.60	1.07	0.35	3.74	0.75	0.49	−1.00
Q14	3.76	0.62	0.19	−0.36	3.74	0.54	−0.17	−0.18
Q15	3.67	0.96	0.02	−0.96	3.82	0.83	−0.68	0.46
PO1	3.52	0.85	0.29	−0.61	3.74	0.82	0.23	−1.14
PO2	3.81	0.83	0.13	−0.54	3.83	0.89	0.32	−1.22
PO3	3.38	0.82	0.04	−0.01	3.43	0.79	0.30	−0.06
PO4	3.62	0.67	0.67	0.28	3.65	0.71	0.13	−0.62
PO5	4.10	0.77	−0.13	−0.76	4.04	0.88	−0.32	−1.22

**Table 4:** The overall differences in students’ attitudes and tutors’ observations at distance/traditional learning modes.

	Levene’s Test for Equality of Variances		*t*-Test for Equality of Means					
	F	Sig.	t	df	Sig. (2-Tailed)	Mean Difference	95% Confidence Interval of the Difference	
							Lower	Upper
With equal var.	1.285	0.263	−6.633	42	**0.000** *	−0.328	0.049	−0.428
Without equal var.			−6.710	40.993	0.000	−0.328	0.048	−0.427

* The main difference is significant at the 0.05 level.

**Table 5:** Tutors’ observations toward students’ programming outcomes.

		Levene’s Test for Equality of Variances		*t*-Test for Equality of Means					
		F	Sig.	t	df	Sig. (2-Tailed)	Mean Difference	95% Confidence Interval of the Difference	
								Lower	Upper
PO1	With equal var.	0.486	0.489	−1.216	42	0.231	−0.250	−0.666	0.165
	Without equal var.			−1.230	41.024	0.226	−0.250	−0.661	0.160
PO2	With equal var.	1.792	0.188	−0.766	42	0.448	−0.159	−0.579	0.260
	Without equal var.			−0.776	40.389	0.442	−0.159	−0.574	0.255
PO3	With equal var.	0.003	0.959	−0.635	42	0.529	−0.140	−0.588	0.306
	Without equal var.			−0.634	41.532	0.529	−0.140	−0.588	0.307
PO4	With equal var.	1.483	0.230	−1.120	42	0.269	−0.223	−0.626	0.179
	Without equal var.			−1.107	37.446	0.275	−0.223	−0.632	0.185
PO5	With equal var.	0.980	0.328	−0.351	42	0.727	−0.078	−0.530	0.373
	Without equal var.			−0.353	41.994	0.726	−0.078	−0.528	0.371

**Table 6:** Students’ attitudes toward satisfaction dimension.

		Levene’s Test for Equality of Variances		*t*-Test for Equality of Means					
		F	Sig.	t	df	Sig. (2-Tailed)	Mean Difference	95% Confidence Interval of the Difference	
								Lower	Upper
Q1	With equal var.	0.003	0.956	−1.951	42	0.058	−0.459	0.235	−0.935
	Without equal var.			−1.950	41.576	0.058	−0.459	0.235	−0.935
Q2	With equal var.	0.324	0.572	−4.503	42	**0.000** *	−0.958	0.212	−1.388
	Without equal var.			−4.478	40.137	0.000	−0.958	0.214	−1.391
Q3	With equal var.	4.105	0.049	−2.687	42	0.010	−0.598	0.222	−1.047
	Without equal var.			−2.643	35.247	**0.012** *	−0.598	0.226	−1.057
Q4	With equal var.	5.846	0.020	−0.600	42	0.874	−0.041	0.258	−0.563
	Without equal var.			−0.157	33.590	0.876	−0.041	0.263	−0.577
Q5	With equal var.	0.072	0.789	−0.990	42	0.328	−0.213	0.215	−0.647
	Without equal var.			−0.987	40.892	0.330	−0.213	0.216	−0.649

* The main difference is significant at the 0.05 level.

**Table 7:** Students’ attitudes toward enjoyment dimension.

		Levene’s Test for Equality of Variances		*t*-Test for Equality of Means					
		F	Sig.	t	df	Sig. (2-Tailed)	Mean Difference	95% Confidence Interval of the Difference	
								Lower	Upper
Q6	With equal var.	5.273	0.027	−1.812	42	0.077	−0.484	0.267	−1.024
	Without equal var.			−1.784	35.856	0.083	−0.484	0.271	−1.035
Q7	With equal var.	1.168	0.286	−2.156	42	**0.037** *	−0.472	0.218	−0.913
	Without equal var.			−2.150	41.088	0.037	−0.472	0.219	−0.915
Q8	With equal var.	0.002	0.964	0.333	42	0.741	0.064	0.192	−0.324
	Without equal var.			0.334	41.881	0.740	0.064	0.192	−0.323
Q9	With equal var.	6.341	0.016	−2.313	42	0.026	−0.598	0.258	−1.120
	Without equal var.			−2.270	34.023	**0.030** *	−0.598	0.263	−1.134
Q10	With equal var.	0.748	0.392	−2.128	42	**0.039** *	−0.536	0.251	−1.044
	Without equal var.			−2.110	39.018	0.041	−0.536	0.254	−1.050

* The main difference is significant at the 0.05 level.

**Table 8:** Students’ attitudes toward motivation dimension.

		Levene’s Test for Equality of Variances		*t*-Test for Equality of Means					
		F	Sig.	t	df	Sig. (2-Tailed)	Mean Difference	95% Confidence Interval of the Difference	
								Lower	Upper
Q11	With equal var.	5.643	0.022	−3.275	42	0.002	−0.807	0.246	−1.305
	Without equal var.			−3.202	31.775	**0.003** *	−0.807	0.252	−1.321
Q12	With equal var.	0.675	0.416	−1.040	42	0.304	−0.207	0.198	−0.608
	Without equal var.			−1.039	41.389	0.305	−0.207	0.199	−0.609
Q13	With equal var.	1.392	0.245	−1.507	42	0.139	−0.310	0.206	−0.726
	Without equal var.			−1.523	41.253	0.135	−0.310	0.203	−0.722
Q14	With equal var.	0.372	0.545	0.130	42	0.898	0.022	0.175	−0.331
	Without equal var.			0.129	39.792	0.898	0.022	0.176	−0.334
Q15	With equal var.	3.234	0.079	−0.392	42	0.697	−0.111	0.285	−0.688
	Without equal var.			−0.387	38.058	0.701	−0.111	0.288	−0.696

* The main difference is significant at the 0.05 level.

## Data Availability

Data are available upon request. Please contact Taras Panskyi (tpanski@iis.p.lodz.pl).
